# The clinical and educational outcomes of an inter-professional student-led medication review team, a pilot study

**DOI:** 10.1007/s00228-020-02972-3

**Published:** 2020-08-08

**Authors:** Michael O. Reumerman, Milan C. Richir, Philippe M. Domela Nieuwenhuis, Rowan Sultan, Hester E. M. Daelmans, Hans Springer, Majon Muller, Michiel A. van Agtmael, Jelle Tichelaar

**Affiliations:** 1 Department of Internal Medicine, Section Pharmacotherapy, Amsterdam UMC, location VUmc, Amsterdam, The Netherlands; 2 Research & Expertise Center In Pharmacotherapy Education (RECIPE), Amsterdam, Netherlands; 3 VUmc School of Medical Sciences, Institute of Education and Training, Amsterdam, Netherlands; 4 Hogeschool Inholland, Amsterdam, Netherlands; 5 Department of Internal Medicine and Geriatrics, Amsterdam UMC, location VUmc, Amsterdam, Netherlands

**Keywords:** Clinical pharmacology, Inter-professional, Medical education, Pharmacotherapy, Medication review, Internal medicine, Elderly care

## Abstract

**Aims:**

The involvement of an inter-professional healthcare student team in the review of medications used by geriatric patients could not only provide patients with optimized therapy but also provide students with a valuable inter-professional learning experience. We describe and evaluate the clinical and learning outcomes of an inter-professional student-run mediation review program (ISP).

**Subject and method:**

A variable team consisting of students in medicine, pharmacy, master advanced nursing practice, and master physician assistant reviewed the medication lists of patients attending a specialized geriatric outpatient clinic.

**Results:**

During 32 outpatient visits, 188 medications were reviewed. The students identified 14 medication-related problems, of which 4 were not recognized by healthcare professionals. The ISP team advised 95 medication changes, of which 68 (71.6%) were directly implemented. Students evaluated this pilot program positively and considered it educational (median score 4 out of 5) and thought it would contribute to their future inter-professional relationships.

**Conclusion:**

An inter-professional team of healthcare students is an innovative healthcare improvement for (academic) hospitals to increase medication safety. Most formulated advices were directly incorporated in daily practice and could prevent future medication-related harm. The ISP also offers students a first opportunity to work in an inter-professional manner and get insight into the perspectives and qualities of their future colleagues.

**Electronic supplementary material:**

The online version of this article (10.1007/s00228-020-02972-3) contains supplementary material, which is available to authorized users.

## Introduction

Medicine is becoming increasingly specialized, with specific referral questions and clearly defined outpatient clinical goals. Even though different physicians and specialists are expected to know the medications their patients use, relatively little attention is paid to the optimization of these medications. Since there are a growing number of elderly patients often using multiple medications, there is an increased risk of adverse drug reactions (ADRs). Therefore, it is essential to review patients’ medication lists in clinical practice [1, 2].

Although medical curricula are devoting more time to pharmacotherapy [3], the teaching of geriatric pharmacotherapy and training in performing a medication review are still not standard practice [4]. A medication review is a complex task, involves various healthcare specialists, and necessitates optimal inter-professional collaboration. When implemented correctly**,** it can improve medication safety by reducing potentially inappropriate medications and potential prescribing **omissions** [5, 6].

To prepare healthcare students for their future role in medication safety, it is important to teach them to learn and work with each other and with other healthcare professionals [7–9]. This is especially important in the assessment of polypharmacy in geriatric patients [10]. Therefore, we developed a context-based, inter-professional, student-led medication review program (ISP), and in this study we investigated its effect on patients’ medication changes and students’ learning outcomes.

## Methods

The inter-professional student-led medication review program (ISP) was developed to systematically analyze the medications of 50% of patients visiting the memory outpatient clinic of the Center of Geriatric medicine Amsterdam (COGA) of the Amsterdam University Medical Centre, location VUmc. The ISP was added on top of standard care, which consisted of a physician evaluating the medication during their consultation. The patient group consisted of patients aged 70 years or older who were suspected of cognitive decline and used on average more than 5 chronic medications (Table 1). Each week, a 3-member team of bachelor and master medical students, pharmacy students, physician assistants, or advanced nursing practice students (equipped with documentation regarding the Prescribing Optimalisation Method (POM) [11] and START-STOPP criteria [12]) evaluated the medication of patients who visited this clinic.

**Table 1:**
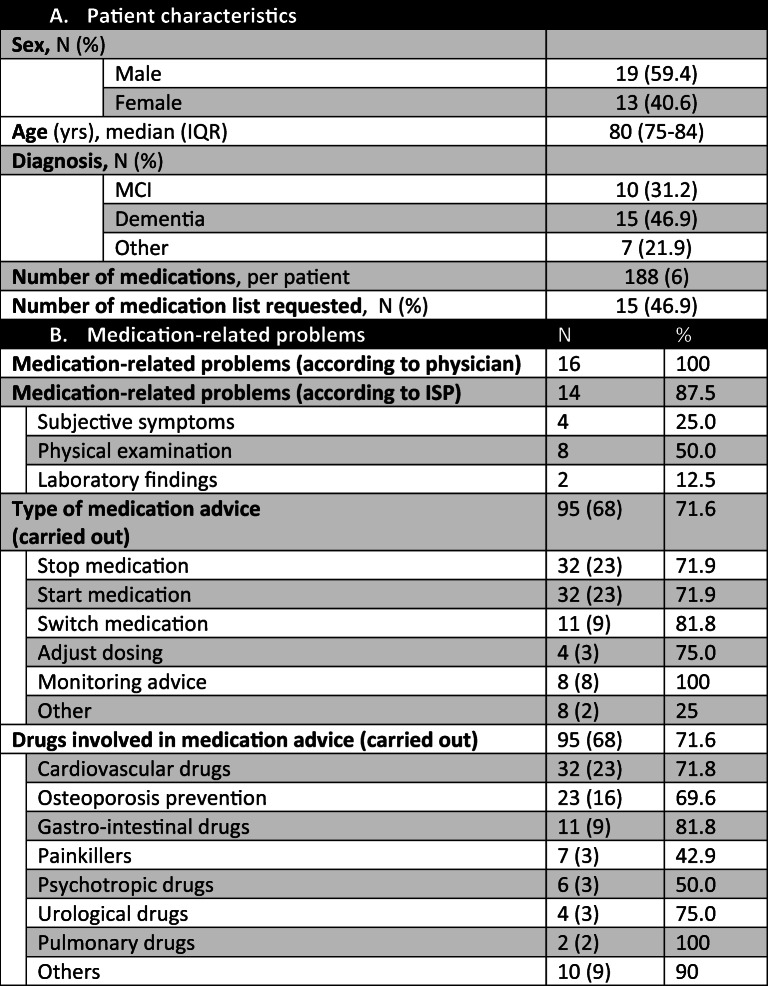
Clinical results. *A*: Characteristics of patients (*n* = 32) evaluated at the memory clinic by the ISP teams. *B*: Medication review outcomes: medication-related problems, type of medication advice, and drugs involved

### ISP team procedure

**Fig. 1:**
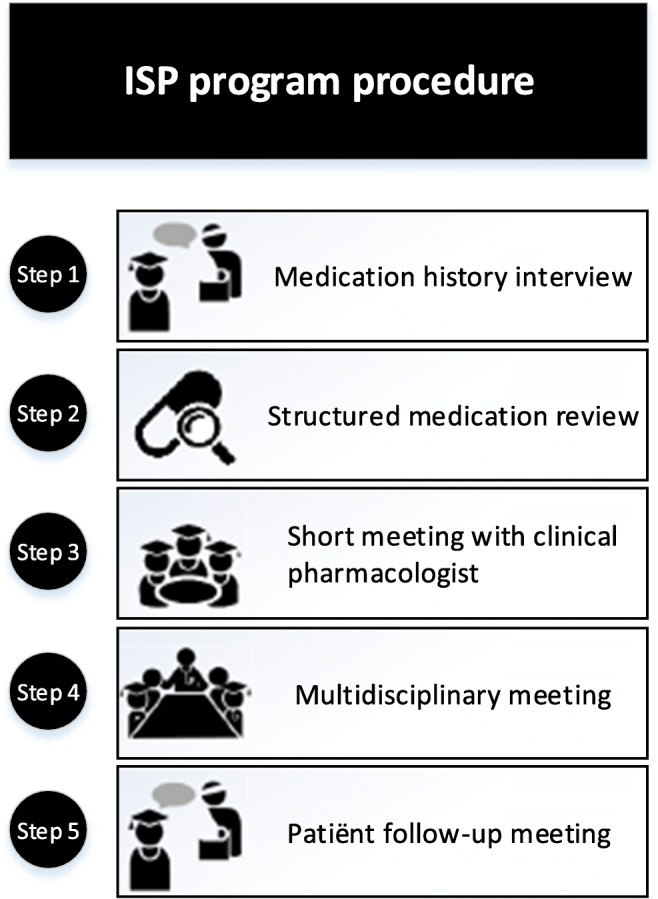
Inter-professional student-run mediation review program (ISP) procedure consisting of 5 steps. *Step 1*: Taking a medication history (30 min) and requesting the medication lists from patients’ pharmacists. *Step 2*: Performing a structured medication review using the Prescribing Optimisation Method (POM) and STOPP/START criteria. *Step 3*: Having a short meeting with a clinical pharmacologist. *Step 4*: Discussing medication advice at a multidisciplinary meeting and updating the electronic patient system. *Step 5*: Explaining the medication changes to patients at a follow-up appointment

The ISP carried out the 5-step ISP program (Fig. 1), consisting of (1) taking a medication history (30 min) with patient and or caregiver/family and requesting medication lists from patients’ community pharmacists; (2) performing a structured medication review using the POM; (3) discussing the review results with clinical pharmacologist (20 min/week, as part of pharmacotherapy education for interns); (4) submitting the proposed medication advice in a multidisciplinary meeting and updating the electronic patient system; and (5) explaining the medication changes to the patient and caregiver/family at a follow-up appointment.

This program is a collaboration between the Learner-Centred Student-Run Clinic (LC-SRC) [13–15], which is part of the Section Pharmacotherapy, and the Department of Geriatric Medicine of Amsterdam University Medical Centre, location VUmc. It is coordinated by a (non-payed) senior healthcare student and was supervised by a clinical pharmacologist and staff of the COGA. The clinical pharmacologist discussed the cases during the (already present) weekly education/training sessions. COGA staff (who were already present at the multidisciplinary meeting) supervised the ISP team as they would other physicians and nurses. The extra time-expenditure during the multidisciplinary meeting, for discussing the additional medication changes on top of standard care, was on average 1 min per patient.

### Evaluation instruments

Medication advice was analyzed by assessing the electronic patient healthcare records. Patient medication lists, and advice about medication changes given by the ISP team and the other healthcare professional (geriatric physician), were anonymously extracted from the records and compared with the medication lists mentioned in outpatient clinic correspondence. Satisfaction and learning outcomes of students were measured using a voluntary digital student survey consisting of 20 multiple-choice and open-ended questions.

### Statistics

Data were analyzed using SPSS Statistics 22 (IBM Corporation, Armonk, NY, USA). Descriptive statistics were computed for the student and patient populations, medication advice, and student outcomes. Student open-ended questions were analyzed using content/thematic analysis.

### Ethical aspects

The institutional review board of VUmc reviewed the protocol and concluded that the study did not fall under the scope of the Dutch Medical Research Involving Human Subjects Act (WMO) (reference: 15.148). As part of a larger study, our protocol was also approved by the ethics review board of the Netherlands Association for Medical Education (NVMO) (ID:2019.2.1).

## Results

From October 2017 to March 2018, the medication of half (*n* = 32) of all patients attending the COGA was reviewed by ISP teams. None of the 32 patients (median age 80 years, men 59.4%) had had their medications reviewed in the previous year. After the outpatient visit, most patients were diagnosed with dementia (46.9%) or mild cognitive impairment (MCI) (31.2%). They used a median number of 6 (25th–75th percentile: 3–8) medications (Table 1).

### Student characteristics

Thirty-eight students participated. These were bachelor medical students (VU University) who participated as part of the LC-SRC VUmc; master medical students who participated during their Internal Medicine internship at the VU University Medical Centre; master pharmacy students of the University of Utrecht who participated as part of their hospital pharmacy internship at the VU University Medical Centre; or second-year advanced nursing practice (ANP) and third-year physician assistants (PA) students studying at the Hogeschool Inholland Amsterdam. Master medical and pharmacy students participated during their internship, all other students participated voluntarily. In total, 34 filled in the post-participation e-questionnaire (89.5%) (Supplementary Table 1).

### Medication-related problems

When taking the patients’ medication history (step 1), the ISP teams identified 14 possible medication-related problems (Table 1). Eight problems were detected during the physical examination by the geriatric physician (mostly orthostatic hypotension and rigidity) and related to medication by the ISP team and two were laboratory abnormalities (hyperkalemia and extremely high vitamin levels). Four problems were subjective symptoms (confusion, myalgia, dyspepsia, and obstipation) and had not been documented or reported earlier by other healthcare professionals. However, the ISP teams failed to address medication-related problems in two patients, namely, rigidity (possibly caused by antipsychotics) and orthostatic hypotension (possibly caused by long-acting nitrates).

### Medication review

The ISP teams asked the patients’ pharmacists to supply 15 medication lists (46.9% of total) because the patients had forgotten to bring their medication lists, the list was too old, or did not correspond with that held in the electronic patient database (Table 1).

In total, 188 medications were reviewed by the students, who proposed 95 changes (Table 1).

**Fig. 2:**
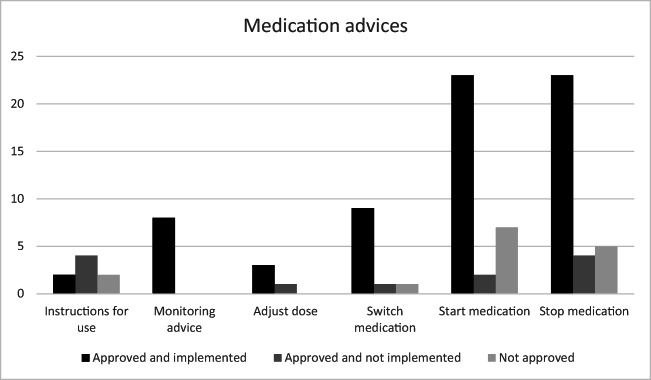
Medication advice after a full medication review categorized by type of advice. *Black*: ISP team advice, approved in multidisciplinary meeting and implemented. *Dark grey*: ISP team advice approved in multidisciplinary meeting but not implemented. *Light grey*: ISP team advice given but not approved in multidisciplinary meeting and not implemented. *Monitor advice*: e.g., monitor blood pressure. *Instructions for use*: e.g., do not chew on drug x

In the multidisciplinary meeting, the proposed changes for 80 medications (84.2%) were accepted by the head of the department of the memory outpatient clinic of the COGA, of which 68 (71.6%) were directly implemented (incorporated in the outpatient letter) (Fig. 2). Most changes were about starting (*n* = 32), stopping (*n* = 32), or switching (*n* = 11) medications (Table 1). Students also evaluated dosing, discrepancies in medication lists, compliance and ingestion, and medication schedules and advised about monitoring medication changes.

Most changes were made to cardiovascular drugs (*n* = 32), drugs used for osteoporosis prevention (*n* = 23), and gastrointestinal drugs (*n* = 11). Psychotropic and urological drugs were mostly presumed to cause the medication-related problems (i.e., confusion and orthostatic hypotension).

### Student results

Most students had previously performed one or more steps of the medication review process, but none had performed all the consecutive steps before participating in the ISP (Table 2A). Students considered all steps of the medication review to be educational (median 4–5, 1–5 min/max) and more interesting than learning to review medications based on constructed patient cases on paper (median 4–5, 1–5 min/max). They agreed that the first steps of the medication review were more difficult in practice than in constructed patient cases (median 4–5, 1–5 min/max), but felt that explaining the medication changes to patients was less difficult (median 3, 1–5 min/max) than the other steps of the process. Students thought the medication review program added value to their curriculum (median score 4, 1–5 min/max) (Table 2B). They rated the program a median of 85 out of 100.

**Table 2:**
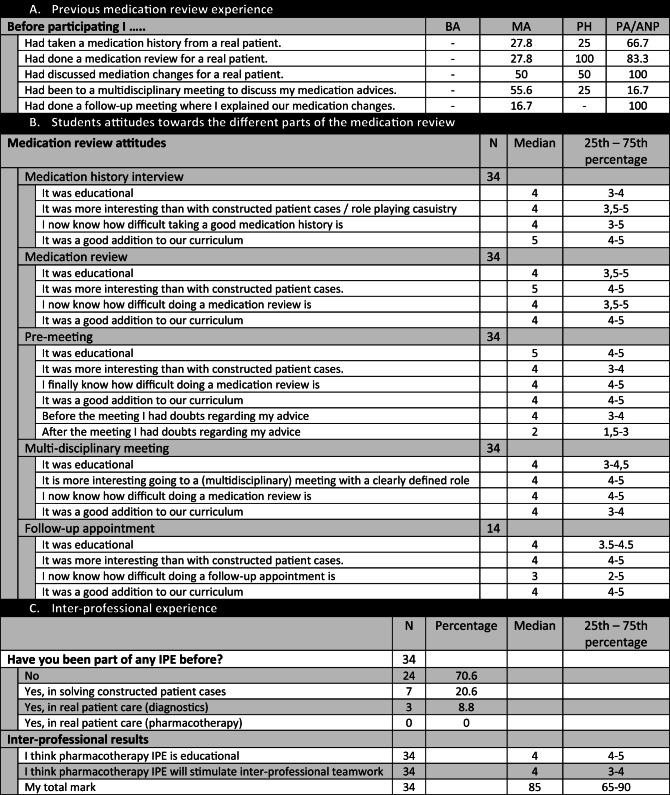
Educational results. *A*: Student medication review experience categorized by student types. *B*: Student attitudes toward the different parts of the medication review (1. medication history interview, 2. medication review, 3. pre-meeting, 4. multidisciplinary meeting, and 5. follow-up appointment). *C*: Inter-professional experience

*BA* medical bachelor students, *MA* medical master students, *PH* pharmacy students, *PA/ANP* physician assistants and advanced nursing practice

### Inter-professional results (quantitative)

Nine students (29.4%) had previously been involved in inter-professional education (IPE), usually solving constructed paper cases on paper, before participating in the ISP. Three students had received IPE with regard to diagnostics involving real patients, but none had received IPE on pharmacotherapy. All students agreed they learned from the inter-professional program (median 4, 1–5 min/max) and that the program would stimulate future inter-professional teamwork (median 4, 1–5 min/max) (Table 2C).

### Inter-professional results (qualitative)

Most students (91%) found working in an inter-professional setting valuable. Pharmacy students were typically considered to have the most knowledge of drug interactions and guidelines (10 comments), but they had limited knowledge about comorbidities (5 comments) and less empathy for older, cognitively impaired patients (4 comments). Medical students were considered to have the best clinical knowledge (10 comments) and were good at making decisions (5 comments), but they were less concerned about medication safety (3 comments). ANP/PA students were considered to be extremely practical, caring with a great knowledge of guidelines (6 comments). Only one student considered ANP/PA students less knowledgeable about disease pathology.

## Discussion

The ISP is not only a unique and innovative learning opportunity for different healthcare students; it might also be a healthcare improvement for (academic) hospitals. Since a medication review is not standard care during outpatient clinic visits, it is a good opportunity to evaluate the value of an (inter-professional) student team (ISP) on top of standard care and potentially increase medication safety The patients who visited the memory clinic were offered a structured medication review without any extra cost to themselves and with only minimal effort for the healthcare system. Of the proposed advices, 84.2% were accepted in the multidisciplinary meeting, of which 85.0% (71.6% of total) was directly incorporated into daily practice (Fig. 2). Students also considered the program to be educational and thought it would contribute to future inter-professional collaboration.

To our knowledge, this is the first study to evaluate the clinical and inter-professional learning possibilities of inter-professional student teams working together to review the medications of patients attending an outpatient clinic. Similar to other studies that focused on IPE, we also found that the students gained an understanding of other health professionals’ capabilities [9, 16, 17]. Since previous studies have shown that healthcare students can actively be involved in taking medication histories and/or giving medication advice [16, 18], our study is the first to show that a trained inter-professional student team can perform all the steps of a full medication review.

The strengths of this study lies in the unique collaboration between medical students, pharmacy students, advanced nursing practice students, and physician assistant students. By learning and working together on real clinical tasks, students encountered each other’s competences, boundaries, and professional responsibilities. Having knowledge of these aspects will help future healthcare providers to collaborate and communicate more effectively, which will benefit patient safety [5]. A second strength is the scalability of this project. Although the pilot project only included one outpatient clinic, more outpatient clinics have been added to our program. With students from the LC-SRC VUmc and interns in abundance, there is no problem to eventually serve most of the outpatient clinics of a university hospital. A third strength is the setting of the outpatient clinic. Patients with presumed cognitive decline are a frail and difficult patient group, and many would benefit from optimization of their medications, either by reducing the number of drugs acting on cognition or by reducing the quantity or dosing frequency of the drugs prescribed. Lastly, even though some students had previously carried out one or more steps of a medication review, here they performed all steps, working as a team. This improves students’ intrinsic motivation and ability to collaborate inter-professionally [19].

A limitation of this pilot study is the single-center design with a relatively small heterogeneous sample size (38 students and 32 patients). Despite this, the response rate for the e-questionnaire was high (89.5%). Another limitation is self-selection bias, which could have played a role for the bachelor medical students and (specialist) nurses who participated voluntarily. Since most students were medical and pharmacy interns to which the program was mandatory (65%), this form of bias will probably be of minor importance.

Taking these strengths and limitations into account, we conclude that an inter-professional team of healthcare students is able to give valuable medication advice to geriatric patients on polypharmacy. This creates a win-win-win situation. Students have learning opportunities with responsibility for real patients while working in an inter-professional setting, as suggested by Brinkman et al. [3], patients receive a full medication check-up, and healthcare professionals can focus on primary outpatient goals. An intervention study to analyze the clinical effects and inter-professional benefits in more detail is in progress.

## Electronic supplementary material

Supplementary table 1 Inter-professional student led medication review program student characteristics. (DOCX 14 kb)

## Data Availability

The data that support the findings of this study are available from the corresponding author upon reasonable request.
