# Supplementary material for: The clinical and educational outcomes of an inter-professional student-led medication review team, a pilot study
Source: Eur J Clin Pharmacol. 2020 Aug 8;77(1):117–23. doi: 10.1007/s00228-020-02972-3 (PMC7782385; doi:10.1007/s00228-020-02972-3)
Supplement: Supplementary file 1 — Inter-professional student led medication review program student characteristics. (DOCX 14 kb) [file 228_2020_2972_MOESM1_ESM.docx]

**Supplemental students results**

Bachelor medical students had never performed a medication review, whereas master students had mostly only discussed medication changes or had actively participated in a multidisciplinary meeting. Pharmacy students had experience in reviewing the medications of real patients but had not completed the other steps of the medication review. PA and ANP students had been involved in most steps although only one student had actively participated in the multidisciplinary meeting **(Table 2A)**.

# Supplementary table 1

| **Student characteristics** | | N | % |
| --- | --- | --- | --- |
| **Sex** | | | |
|  | Male | 15 | 44.1 |
|  | Female | 19 | 55.9 |
| **Student type** | | | |
|  | Bachelor medicine | 6 | 17.6 |
|  | Master medicine (internship) | 18 | 52.9 |
|  | Pharmacy | 4 | 11.8 |
|  | Physician assistant | 4 | 11.8 |
|  | Advanced nursing practice | 2 | 5.9 |

**Supplementary table 1.** Inter-professional student led medication review program student characteristics.
